# Transcriptome and Low-Affinity Sodium Transport Analysis Reveals Salt Tolerance Variations between Two Poplar Trees

**DOI:** 10.3390/ijms24065732

**Published:** 2023-03-17

**Authors:** Xuan Ma, Qiang Zhang, Yongbin Ou, Lijun Wang, Yongfeng Gao, Gutiérrez Rodríguez Lucas, Víctor Resco de Dios, Yinan Yao

**Affiliations:** 1 School of Life Science and Engineering, Southwest University of Science and Technology, Mianyang 621010, China; 2 Department of Crop and Forest Sciences & Agrotecnio Center, Universitat de Lleida, 25003 Leida, Spain

**Keywords:** salt stress, *Populus*, Na^+^ transportation, transcriptome, HKT1;2

## Abstract

Salinity stress severely hampers plant growth and productivity. How to improve plants’ salt tolerance is an urgent issue. However, the molecular basis of plant resistance to salinity still remains unclear. In this study, we used two poplar species with different salt sensitivities to conduct RNA-sequencing and physiological and pharmacological analyses; the aim is to study the transcriptional profiles and ionic transport characteristics in the roots of the two *Populus* subjected to salt stress under hydroponic culture conditions. Our results show that numerous genes related to energy metabolism were highly expressed in *Populus alba* relative to *Populus russkii*, which activates vigorous metabolic processes and energy reserves for initiating a set of defense responses when suffering from salinity stress. Moreover, we found the capacity of Na^+^ transportation by the *P. alba* high-affinity K+ transporter1;2 (HKT1;2) was superior to that of *P. russkii* under salt stress, which enables *P. alba* to efficiently recycle xylem-loaded Na^+^ and to maintain shoot K^+^/Na^+^ homeostasis. Furthermore, the genes involved in the synthesis of ethylene and abscisic acid were up-regulated in *P. alba* but downregulated in *P. russkii* under salt stress. In *P. alba,* the gibberellin inactivation and auxin signaling genes with steady high transcriptions, several antioxidant enzymes activities (such as peroxidase [POD], ascorbate peroxidase [APX], and glutathione reductase [GR]), and glycine-betaine content were significantly increased under salt stress. These factors altogether confer *P. alba* a higher resistance to salinity, achieving a more efficient coordination between growth modulation and defense response. Our research provides significant evidence to improve the salt tolerance of crops or woody plants.

## 1. Introduction

Soil salinity is a detrimental environmental stress that constrains plant growth and crop production. High salinity induces osmotic stress, ionic toxicity, and oxidative stress in plants [1,2]. Saline soils limit the capacity of plant roots to absorb water, promoting water-deficiency and osmotic stress, which results in the inhibition of plant growth. Furthermore, when an excess of sodium (Na^+^) and/or chlorine (Cl^−^) ions enter through the transpiration stream into the plant leaves, it causes ionic imbalance and cellular damage, hampering plant growth and development [3,4]. These osmotic and ionic stresses may induce further secondary oxidative effects, promoting retardation of plant growth, metabolic and developmental changes, and reduction of productivity [1,4,5].

The transport of Na^+^ into plant roots presents a biphasic behavior [6]: when external Na^+^ concentration is at a very low level, Na^+^ transport is mediated by a high-affinity transport system (HATS); at higher Na^+^ concentrations, it is mediated via a low-affinity system (LATS). Extensive research of the adverse effects of high salinity on agricultural production has, during the last decades, consolidated LATS as a well-acknowledged mechanism in the field of plant physiology [7,8,9]. Electrophysio- and pharmacological analyses have long demonstrated that the LATS of Na^+^ uptake in glycophytes or halophytes (e.g., *Arabidopsis* and *Suaeda maritime*) mainly consist of non-selection cation channels (NSCC) [7,9] as well as several Na^+^/K^+^ transporters, i.e., the low-affinity cation transporter 1 (LCT1), high-affinity K^+^ transporter (HKT), and K^+^ channels *Arabidopsis* K^+^ Transporter 1 (AKT1) [8,9,10,11,12]. HKT1 mediates Na^+^ transport in both high- and low-affinity systems and may function as either an Na^+^ uniporter or Na^+^/K^+^ symporter [4,13]. In *Arabidopsis*, there is only evidence of one HKT ortholog gene *AtHKT1;1* performing Na^+^ exclusion from leaves and regulating Na^+^ distribution between roots and shoots [14,15]. In other plant species, a great number of studies have also revealed that the HKT1 ortholog protein plays a key role in salt tolerance in both dicots and monocots via Na^+^ translocation [15,16,17]. Although the function of HKTs in herbs or grass plants has been thoroughly studied, however, little is yet known about their function in perennial woody plants.

Poplars are widely distributed across the northern temperate and cold-temperate zones of the world. Different *Populus* species evolutionarily adapted to these diverse ecological environments have thus generated long-term variations in responsiveness and tolerance to salinity [18,19,20]. Differences in salt tolerance between *Populus* species possibly result from different Na^+^ transporting or translocation capacities in the plant organ/tissue or cell, including the following: (1) Na^+^ and/or K^+^ uptake capacities are clearly different between salt-tolerant and salt-sensitive *Populus* species under salinity stress [18,19]. (2) Excess Na^+^ remains in roots rather than translocating into the salt-sensitive photosynthetic tissues, which is an efficient strategy to tolerate salinity stress in plants [1,2]. For example, the AtHKT1;1 in *Arabidopsis* and HKT1;5 in rice or wheat perform a similar function in the storge of Na^+^ in roots, unloading Na^+^ from the xylem sap into the surrounding parenchyma cells and thereby protecting the photosynthetic organs from Na^+^ toxicity [14,15,16,21]. (3) Differences are present in the Na^+^ compartmentation and Na^+^ detoxification in cells subjected to salinity stress. The sequestration of Na^+^ within the vacuole through the Na^+^/H^+^ antiporters (NHXs) and the extrusion of Na^+^ from the cytoplasm through the plasma membrane-localized Na^+^/H^+^ antiporter SOS1 (Salt Overly Sensitive) are key mechanisms to improve plants’ salt tolerance at cellular levels [2,22,23].

Moreover, the capacity of K^+^ uptake and maintaining the K^+^/Na^+^ ratio in plant cells is crucial to improve salt tolerance [18,19]. Since Na^+^ competes with K^+^ and induces K^+^ efflux from cytoplasm, affecting various enzymatic processes and metabolic pathways, it can cause nutrient ion imbalance [1,24]. Our recent study shows that maintaining the K^+^/Na^+^ ratio plays a vital role against salinity stress under different nitrogen levels [25]. As Na^+^ enters into the plant cell, K^+^/Na^+^ ratio maintenance is an essential route to tolerate salt-induced osmotic stress to keep water availability inside the cytoplasm via concerted regulation of metabolic processes and strengthened biosynthesis of osmoprotectants such as proline, glycine-betaine, etc. [1,2,26]. Furthermore, excess Na^+^ triggers the generation of reactive oxygen species (ROS) inside the plant cell; the capacity to activate prompt redox responses and enhance the activity of antioxidant enzymes that scavenge the excess ROS is paramount to resist oxidative stress induced by high salinity [1,27]. 

Na^+^ transportation under high salinity conditions plays an important role in plant growth and development [2,18]. However, still little is known about Na^+^ transport and the underlying molecular processes of salt tolerance in poplar plants. The regulation of Na^+^ transport mainly depends on the transcriptional level, which is energy-saving and highly plastic, and also depends on the capacity of Na^+^/K^+^ transporters and related ion channels. In this study, we performed RNA-sequencing and combined the pharmacological analysis with a hydroponic-cultivation condition to analyze the Na^+^ transportation and root transcriptomic profiles in two *Populus* species (*Populus alba* and *Populus russkii*, separately belong to the Leuce section and the Aigeiros section in the *Populus* genus). Our results show distinct features of Na^+^ transportation and extensive variations in physiological and molecular responses to salt stress between the two *Populus*. 

## 2. Results

### 2.1. Na^+^ and K^+^ Uptake in the Two Populus and Their Distinct Physiological Responses to Salt Stress

**Figure 1:**
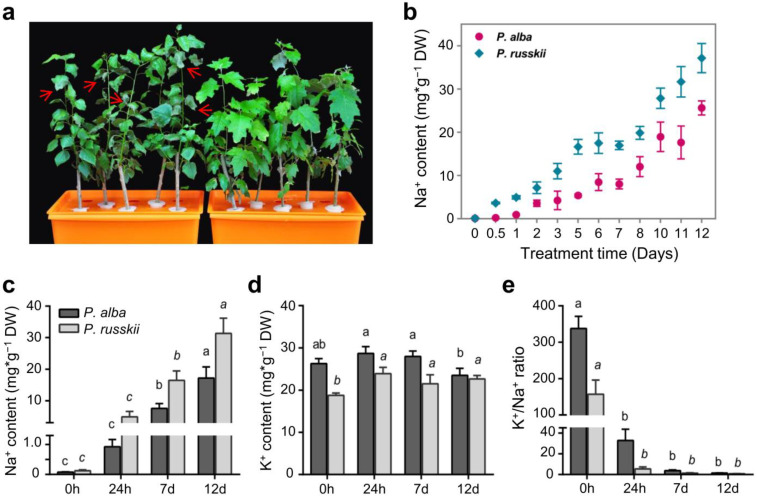
Phenotype and the content of Na^+^ or K^+^ in leaves of two *Populus* species under salt stress. (**a**) Morphological change of *P. russkii* (**left**) and *P. alba* (**right**) with 100 mM NaCl treatment for 12 days. Red arrows indicate the shrunken leaves of *P. russkii*. (**b**) Dynamic of Na^+^ content in the two *Populus* leaves following NaCl treatment. (**c**) Na^+^ content, (**d**) K^+^ content, and (**e**) K^+^/Na^+^ ratio in leaves of *P. alba* and *P. russkii* before treatment and after treatment for 24 h, 7 d, and 12 d. Data shown are the mean ± SD (n = 3). Different letters above the bars indicate the statistical significance at the *p* < 0.05 level among different time-points of NaCl treatment according to LSD test.

To analyze whether there were differences in salt tolerance between *P. alba* and *P. russkii* and explore the characteristics of ion uptake/transport under salinity stress, we cultivated the cuttings of the two poplars using the hydroponic technique (see methods section) and subsequently treated them with 100 mM NaCl. We analyzed the dynamics of Na^+^ and K^+^ contents in the two *Populus* leaves before NaCl treatment (0 h) and at three treatment time points during the treatment (for 24 h, 7 days, and 12 days). Firstly, we detected that Na^+^ accumulation in *P. russkii* leaves was significantly higher than that of *P. alba* (Figure 1b,c), albeit Na^+^ concentration gradually increased in both species during the NaCl treatment. Secondly, K^+^ content was higher in *P. alba* than in *P. russkii* (Figure 1d), while the K^+^/Na^+^ ratio in *P. alba* was also significantly higher than in *P. russkii* (Figure 1e). Thirdly, we observed severe Na^+^ toxicity and osmotic stress in the leaf phenotype of *P. russkii*, whose leaves were dehydrated and shrunk after the NaCl treatment for 12 days, whereas no obvious variation was observed in *P. alba* (Figure 1a). These results altogether indicate that there was a substantial interspecific divergence in salt tolerance and Na^+^ and K^+^ transport between *P. alba* and *P. russkii*. 

### 2.2. Salt Stress Effects on the Antioxidant System of the Two Populus

**Figure 2:**
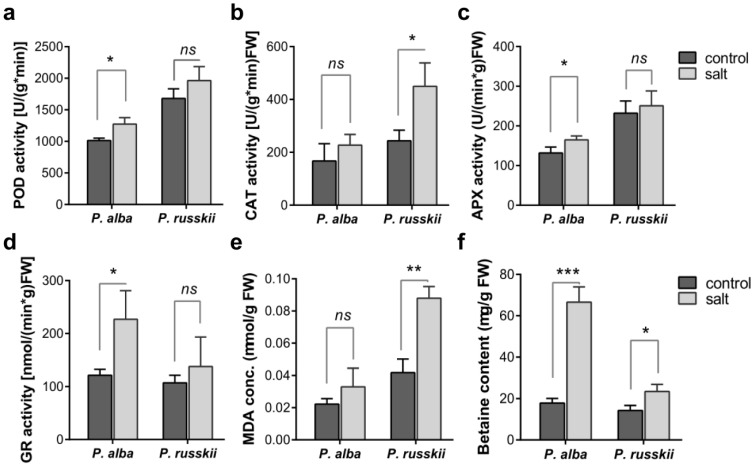
Antioxidant enzyme activities, MDA concentration, and glycine-betaine content in leaves of the two *Populus* species under salinity stress. (**a**) POD activity, (**b**) CAT activity, (**c**) APX activity, (**d**) glutathione reductase (GR) activity, (**e**) MDA concentration and (**f**) glycine-betaine content in *P. alba* and *P. russkii* leaves under NaCl treatment for 12 days. Bar indicates the mean ± SD (n = 3), *, *p* < 0.05, **, *p* < 0.01, ***, *p* < 0.001, ns, *p* > 0.05, two-tailed *t*-test.

The activities of four anti-oxidative enzymes (POD, catalase [CAT], APX, and GR) were determined in the two *Populus* leaves under salt stress (Figure 2a–d). In contrast to the control, POD, APX, and GR activities significantly increased in *P. alba* under salt stress, but no significant changes were found for *P. russkii*; meanwhile, the CAT activity in the two *Populus* showed the opposite trend to the abovementioned three enzymes (Figure 2b). Moreover, malondialdehyde (MDA) concentration significantly increased in *P. russkii* leaves under salt stress but did not increase in *P. alba* (Figure 2e), while glycine-betaine content augmented significantly in both *Populus* leaves under salt stress and increased ca. four times in *P. alba* (Figure 2f). 

### 2.3. Differentially Expressed Genes (DEGs) in P. alba and P. russkii Roots under Salt Stress

**Figure 3:**
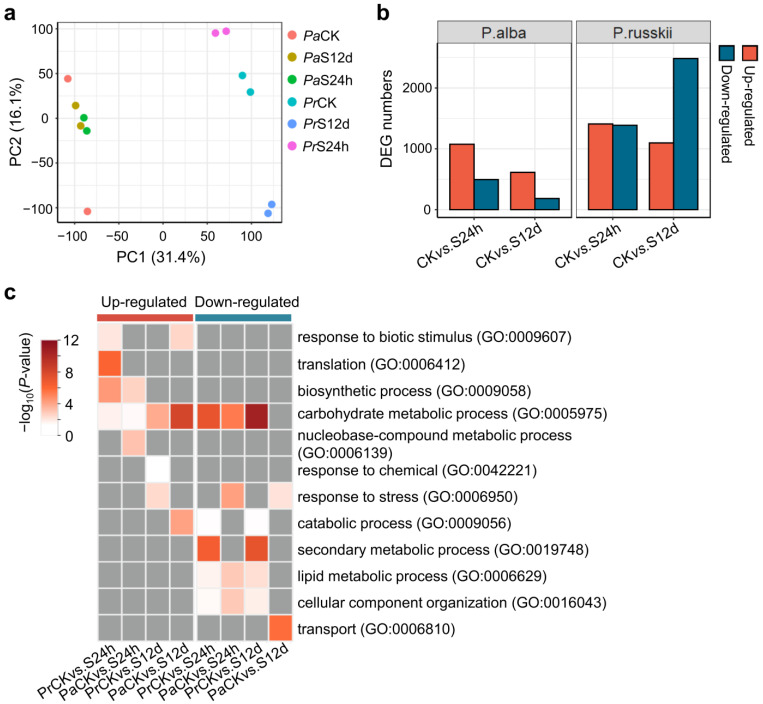
Differentially expressed genes (DEGs) identified in root of *P. alba* and *P. russkii* under salinity stress. (**a**) PCA analysis of the transcriptome data. (**b**) DEG numbers of the identified in *P. alba* and *P. russkii* under NaCl treatment for 24 h and 12 d versus to the control (CK), respectively. (**c**) GO enrichment of the up- and down-regulated genes identified in *P. alba* and *P. russkii* under NaCl treatment for 24 h or 12 d. Grey color indicates not available.

To investigate the molecular basis of the divergent salt tolerance in the two *Populus*, we conducted RNA-sequencing on the roots of *P. alba* and *P. russkii* just after applying the short-term (24 h) and long-term (12 d) salt stress treatments (100 mM NaCl). Two biological replicates were sequenced for each treatment group and control. We obtained an average of 23.3 million reads from twelve cDNA libraries. After removing the adapters and low-quality reads, we obtained high-quality clean reads for each library (Table S1). The clean reads were aligned on the reference genome (v4.1, PhytozomeV13) of *P. trichocarpa*, the reference model poplar species from *Populus* genus; the average mapping rate of *P. russkii* was 74.85% that higher than that of *P. alba* (60.6%), suggesting that the genetic relationship between *P. russkii* and *P. trichocarpa* was closer than that between *P. alba* and *P. trichocarpa*. The numbers of the expressed transcripts (RPKM >1) detected in each library were comparable (average of 22,391, Table S1). The correlation of the two biological replicates for each treatment group was high (Pearson’s *r* = 0.8~1.0, Figure S1). Principal component analysis (PCA) revealed important differences between *P. alba* and *P. russkii* transcriptomes on PC1 (31.4% of variation, Figure 3a). With regard to PC2 (16.1% variation), we observed that the transcriptome of short- and long-term salt stress groups and CK was clearly separated in *P. russkii*, whereas it was not observed in the case of *P. alba* (Figure 3a). One control sample of *P. alba* was far away from other samples, which was attributed to a batch effect of RNA-seq, and therefore was removed for further analysis.

DEGs were identified in *P. alba* and *P. russkii* using a cutoff of fold change ≥ 2 and FDR < 0.01. The numbers of up-regulated genes were more than double those of downregulated genes in the *P. alba* root under either short- (CK (Control) vs. S24h, 1070/493) or long-term salt stress (CK vs. S12d, 609/179) (Figure 3b; Supplementary Data S1). In the *P. russkii* root, the number of up-regulated genes was comparable with that of downregulated genes under short-term salt stress, whereas downregulated genes were two times higher than up-regulated genes under long-term salt stress (2482/1090) (Figure 3b; Supplementary Data S1).

GO enrichment analysis of the up- or downregulated genes in four comparisons (*Pr*CK vs. *Pr*S24h, *Pr*CK vs. *Pr*S12d, *Pa*CK vs. *Pa*S24h, and *Pa*CK vs. *Pa*S12d) identified several biological processes uniquely/commonly overrepresented (Figure 3c). The GO term of the carbohydrate metabolic process (GO:0005975) was significantly enriched in nearly all comparisons (except for downregulated genes in *Pa*CK vs. *Pa*S12d). Several other metabolic processes were significantly enriched in the downregulated genes in *P. russkii* and *P. alba*, such as catabolic (GO:0009056), secondary metabolic (GO:0019748), and lipid metabolic (GO:0006629) processes (Figure 3c). The GO term of response to stress (GO:0006950) was enriched in the up-regulated genes in *P. russkii* (*Pr*CK vs. *Pr*S12d) but in the downregulated genes in *P. alba* (*Pa*CK vs. *Pa*S24h and *Pa*CK vs. *Pa*S12d) (Figure 3c). 

**Figure 4:**
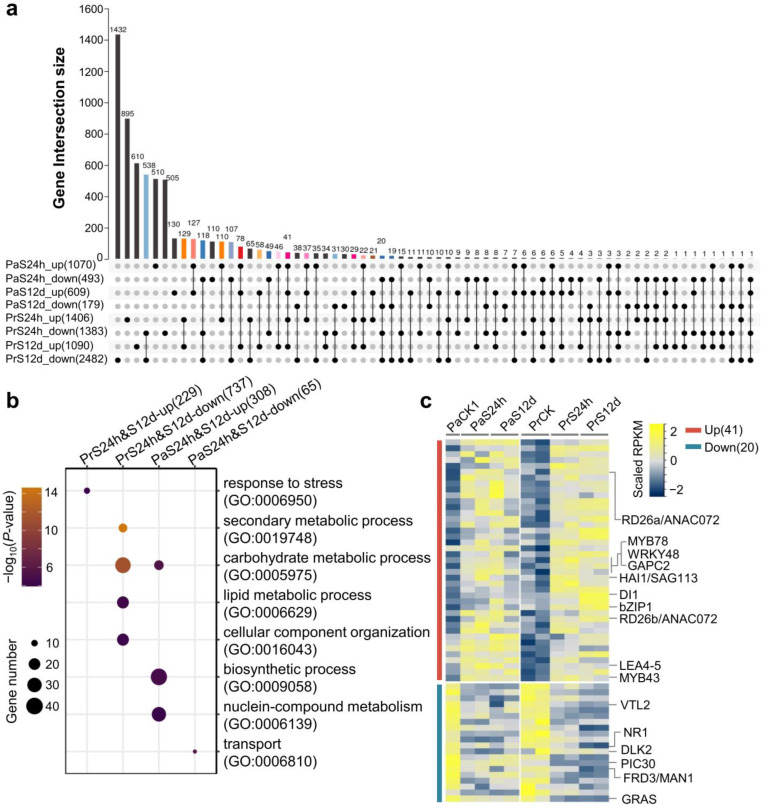
Characteristics of the DEGs identified in *P. russkii* and *P. alba* roots under salt stress. (**a**) Upset-plot showing the overlapping genes among the up-regulated and downregulated genes identified in *P. russkii* and *P. alba* roots. (**b**) GO enrichment of the overlapping up-regulated or downregulated genes of *P. russkii* and *P. alba* roots between short-term and long-term salt stress. (**c**) Heatmap showing the commonly up-regulated or downregulated genes in *P. russkii* and *P. alba* roots under salt stress. Two biological replicates are shown except for the control of *P. alba*.

To further dissect the characteristics of gene transcription in *P. russkii* and *P. alba* roots under salt stress, we analyzed the overlapping of DEGs that were identified in the four comparisons. We found that numerous DEGs overlapped between the short-term and long-term salt stress comparisons in *P. russkii*. For instance, 737 downregulated genes overlapped in *Pr*CK vs. *Pr*S24h and *Pr*CK vs. *Pr*S12d, and 329 up-regulated genes were also overlapped (Figure 4a). The 737 downregulated genes were significantly enriched in response to stress, while the 329 up-regulated genes were mainly enriched in metabolic processes (GO:0019784, GO:0005975, and GO:0006629) (Figure 4b). A total of 308 up-regulated genes overlapped in *Pa*CK vs. *Pa*S24h and *Pa*CK vs. *Pa*S12d that were significantly enriched in carbohydrate metabolic, biosynthetic, and nucleobase-containing compound metabolic processes. A total of 65 downregulated genes overlapped in *Pa*CK vs. *Pa*S24h and *Pa*CK vs. *Pa*S12d that were enriched for transport (Figure 4b). 

Moreover, we found 41 genes were commonly up-regulated in *P. alba* and *P. russkii* under salt stress (Figure 4c), including some transcription factors/genes that were important for response to osmotic stress, abscisic acid, or abiotic stress, such as MYB43/78, WRKY48, bZIP1, Responsive to Dessication 26 (RD26a/b), Highly ABA-Induced PP2C Gene 1 (HAI1), Drought-Induced 21 (DI21), and Late Embryogenesis Abundant 4-5 (LEA4-5) [28,29,30,31,32]. There were 20 genes commonly downregulated in *P. alba* and *P. russkii* under salt stress, including several that were previously reported to play an important role in ion homeostasis and root development, such as Vacuolar iron Transporter-Like 2 (VTL2), Ferric Reductase Defective 3 (FRD3), Nitrate Reductase 1 (NR1), DWARF14-LIKE2 (DLK2), and GRAS [33,34,35,36].

### 2.4. Extensive Variation in Gene Transcription between the Two Populus under Salt Stress 

**Figure 5:**
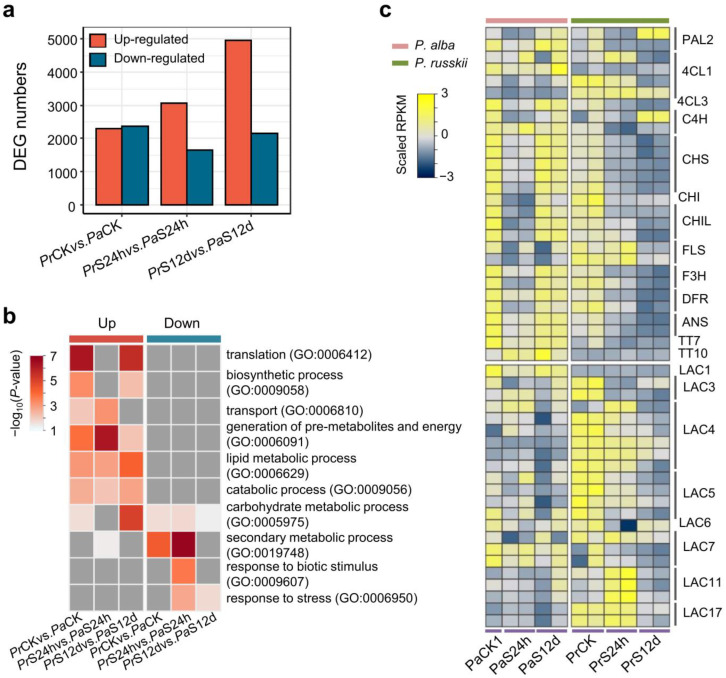
DEGs identified between *P. alba* and *P. russkii*. (**a**) The numbers of DEGs identified between the two *Populus* roots. (**b**) GO enrichment of the up-regulated or downregulated genes between the two *Populus*. (**c**) Heatmap showing the differential expression patterns of the genes in phenylpropanoid pathway and Laccases encoding genes between two *Populus*.

We identified a large number of DEGs between *P. russkii* and *P. alba* in CK and under both short-term and long-term salt stress (Figure 5a; Supplementary Data S2). Numbers of up-regulated and downregulated genes were comparable in CK (*P. russkii* vs. *P. alba*), but under salt stress the up-regulated genes were about two times greater than downregulated genes, suggesting a substantial difference in gene expression between the two *Populus* under salt stress. In the different comparisons for these genes, GO enrichment indicated that up-regulated genes were mainly involved in metabolic and cellular processes, such as generation of precursor metabolites and energy (GO:0006091); lipid metabolic (GO:0006629), catabolic (GO:0009056), and carbohydrate metabolic (GO:0005975) processes; translation (GO:0006412); biosynthetic process (GO:0009058); and transport (GO:0006810) (Figure 5b). The downregulated genes were significantly enriched in metabolic processes (GO:0005975, GO:0019748), response to stress (GO:0006950), and response to biotic stimulus (GO:0009607) (Figure 5b). We found 1080 high-expressing genes and 598 low-expressing genes in *P. alba* versus *P. russkii* regardless of whether they were in CK or under salt stress (Figure S2a,b). These genes might be the interspecific expression genes in the two *Populus*. 

Since the metabolism-related GO term was the most enriched among the DEGs between the two *Populus*, thereafter we analyzed the expression patterns of the genes corresponding to the phenylpropanoid pathway and secondary metabolism. Heatmap analysis showed that most of the key enzyme genes in flavonoids biosynthesis were differentially expressed between the two *Populus* (Figure 5c). Under short-term salt stress, most of these genes were commonly downregulated in the two *Populus*. In addition, we found that many laccases (LAC) encoding genes involved in lignin biosynthesis were differentially expressed in the two *Populus*, i.e., *AtLAC3*, *AtLAC4*, and *AtLAC5* homolog genes being up-regulated in *P. russkii* compared with *P. alba* (Figure 5c). Under short-term salt stress, the homolog genes of *AtLAC4*, *AtLAC11,* and *AtLAC17* were up-regulated in *P. russkii*, suggesting these genes were associated with the rapid response to salt stress in *Populus*. 

**Figure 6:**
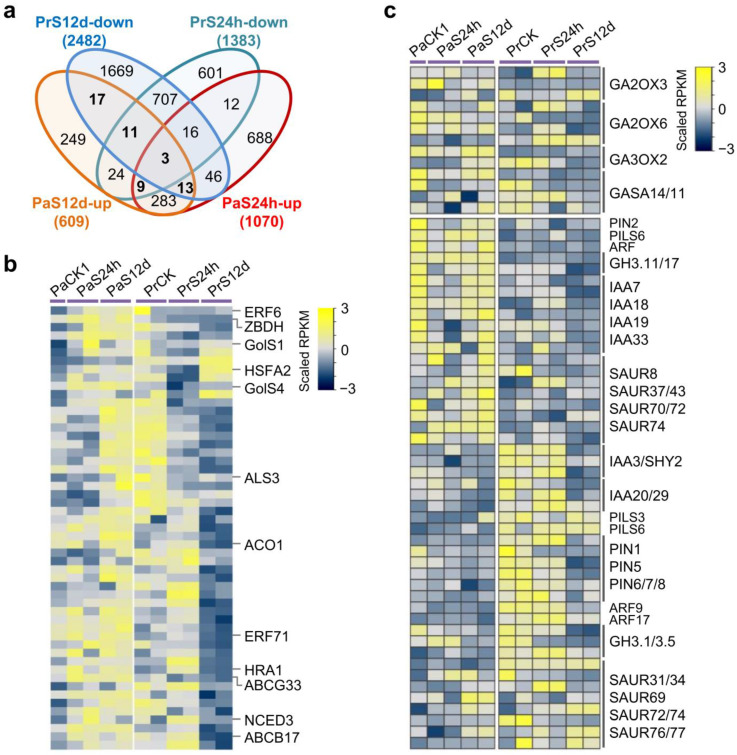
Identification of the expression pattern divergent genes in *P. alba* and *P. russkii* roots under salt stress. (**a**) Venn diagrams showing the overlapping of the up-regulated genes in *P. alba* and downregulated genes in *P. russkii* under salt stress. (**b**) Heatmap showing the genes that the expression patterns were reversed in the two *Populus* under salt stress. (**c**) Heatmap showing the expression of the genes involved in GA or auxin signaling pathways in the two *Populus* under salt stress.

We further analyzed the DEGs identified between the two *Populus*. Interestingly, 151 genes that were up-regulated in the *P. alba* root but were downregulated in *P. russkii* under salt stress (Figure 6a). Among these genes, several had been previously reported to function in response to oxidative stress or abiotic stress (*ERF6, ERF71, ZBDH, HSFA2, GOLS1/4,* and *HRA1*) [37,38,39], hormone biosynthesis (*ACO1* and *NCED3*) [40,41], and ion transport (*ALS3, ABCG33*, and *ABCB17*) (Figure 6b) [42,43]. Furthermore, we analyzed the transcription of the genes in the GA and auxin biosynthetic or signaling pathways, finding substantial differences between the two *Populus* and even presenting a completely reverse pattern; specifically, *GA2OX*s, a GA decomposition gene family, maintained a higher level in *P. alba* under both control and salt stress conditions relative to *P. russkii*. On the other hand, many Auxin/indole-acetic acid (Aux/IAA) repressors (*IAA7, IAA18, IAA19, IAA33*), small auxin up-regulated RNA (SAUR) genes (*SAUR8, SAUR37, SAUR43, SAUR70, SAUR72, SAUR74*), *GH3.11*, *PIN2,* etc., exhibited higher expression levels in *P. alba* than *P. russkii*, both in control and salinity conditions, whereas other IAA genes (*IAA3, IAA20, IAA29*), *PINs* (*PIN1, PIN5-8*), *ARF9, ARF17,* and *GH3.1* were more highly expressed in *P. russkii* than in *P. alba* (Figure 6c).

### 2.5. Differential Expression Patterns of the Na^+^/K^+^ Transporters between the Two Populus under Salt Stress, and Low-Affinity Na^+^ Transport Analysis

**Figure 7:**
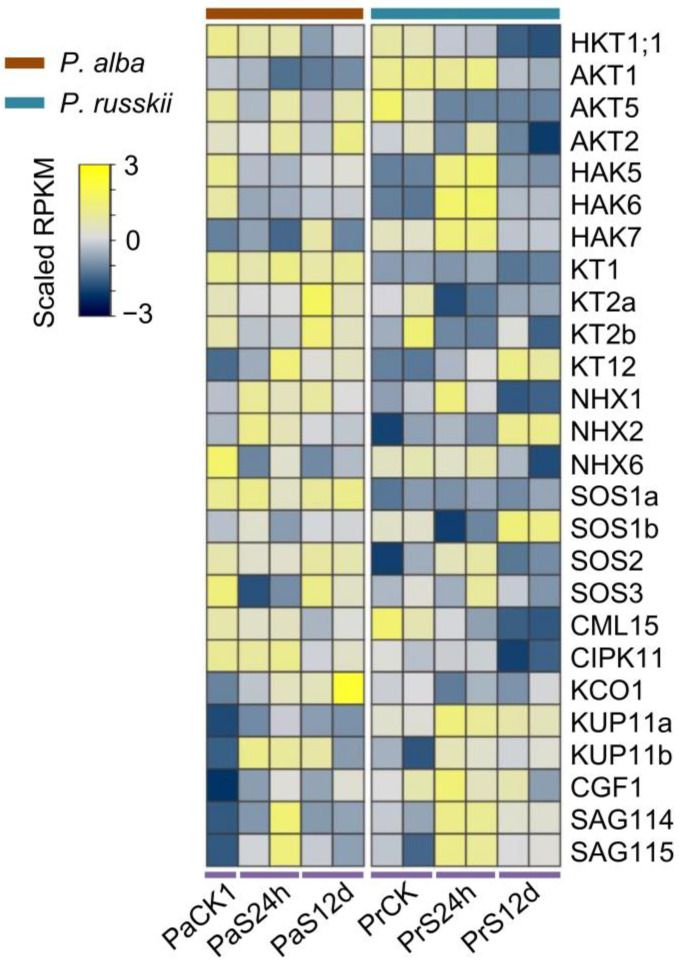
Heatmap showing the different expression patterns of the genes related to Na^+^ or K^+^ transportation in *P. alba* and *P. russkii* roots under salt stress. Two biological replicates were shown except for the control of *P. alba*.

We analyzed the transcription level of Na^+^/K^+^ transporters in *P. alba* and *P. russkii* root transcriptomes, the genes showing distinct expression patterns between the two *Populus* under salt stress (Figure 7). For instance, the Na^+^ transporter gene *HKT1;1* (Potri.018G132200) was significantly downregulated in the *P. russkii* root under salt stress but did not change in *P. alba* under short-term salt stress. Many K^+^ channels (AKT5, AKT2, KT1, KT2a/b, KCO1) and calcium-binding protein (CML15, CIPK11) encoding genes were downregulated in *P. russkii* under salt stress, whereas they did not change in *P. alba* (Figure 7). Furthermore, the transcription levels of Na^+^/H^+^ exchanger genes (NHX1/2/6, SOS1a, SOS2/3) in the *P. alba* root had a higher expression than in *P. russkii*; meanwhile, under long-term salt stress some of them were downregulated in *P. russkii* but did not change in *P. alba* (Figure 7). We designed gene-specific primers for the above transporter genes (*HKT1;1*, *AKT1*, *NHX1/2/3*, *SOS1*, *CBL9*, and *CIPK23/25*) and conducted quantitative real-time PCR (qRT-PCR) analysis to detect their transcription levels and verify the transcriptome data (Figure S4, Table S2). The qRT-PCR results were consistent with the transcriptome data. This confirmed our transcriptome data are accurate and reliable. 

To investigate the Na^+^ uptake/transport features of the two *Populus* under salt stress, we conducted a pharmacological analysis of the low-affinity transport system by the inhibitors of the K^+^ channels (tetraethylammonium chloride [TEA^+^], Cs^+^, and Ba^2+^), Na^+^/K^+^ transporter (Ba^2+^), and non-selective cation channels (Gd^3+^ and La^3+^) [8,9,44]. The results show that Ba^2+^ had strongly inhibited Na^+^ uptaking in *P. alba* but less so in *P. russkii*, that the NSCCs blockers (Gd^3+^ and La^3+^) largely constrained Na^+^ uptake in both *Populus*, and that the K^+^ channel blockers severely inhibited Na^+^ uptaking in *P. alba* more than in *P. russkii* (Table 1). TEA^+^ is a specific K^+^ inward rectifier channels blocker, and its suppression rate in the two *Populus* show that K^+^ channels play a dominant role in Na^+^ transport. Its higher inhibition in *P. alba* than in *P. russkii* suggests the K^+^ channels of *P. alba* have a stronger capacity than those of *P. russkii*. The inhibition of TEA^+^ from Ba^2+^ (i.e., Na^+^ transportation via HKT transporter), which shows a higher suppression value in *P. alba* than *P. russkii*, reveals that *P. alba* HKT has superior Na^+^ transportation than *P. russkii* (Table 1). 

**Figure 8:**
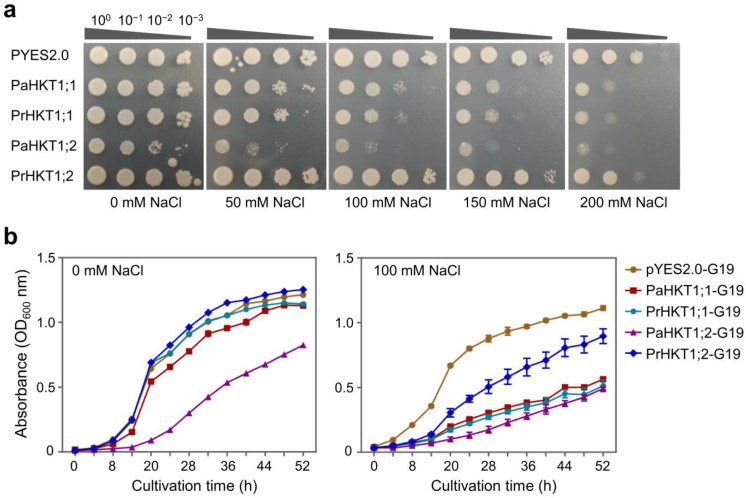
Difference in the Na^+^ transport function of the HKT1;1 and HKT1;2 in *P. alba* and *P. russkii*. (**a**) Yeast mutant G19 complementary analysis of the Na^+^ transport function of PaHKT1;1, PrHKT1;1, PaHKT1;2, and PrHKT1;2. (**b**) Yeast growth curve of the transformants of PaHKT1;1, PrHKT1;1, PaHKT1;2, and PrHKT1;2 to G19 in medium with 100 mM NaCl or without NaCl.

Based on the pharmacological analysis and transcriptome data, we infer that the HKT function of *P. alba* may be divergent from that of *P. russkii*. To confirm this hypothesis, we cloned the *HKT1;1* and *HKT1;2* from *P. alba* and *P. russkii* and obtained the full-length coding sequences. The alignment of the *HKT1;1* and *HKT1;2* sequences of the two *Populus* showed extensive SNP variations (Figures S5 and S6). To study whether these sequence variations affected or not the function of Na^+^ transporting, we conducted an additional Na^+^ transport analysis by separately transforming the *PrHKT1;1*, *PaHKT1;1*, *PrHKT1;2,* and *PaHKT1;2* to complement the yeast Na^+^ transport loss-function mutant G19 (Figure 8a). The yeast growth curves showed that the function of PaHKT1;1 and PrHKT1;1 presented no difference, while the Na^+^ transportation of PaHKT1;2 was significantly higher than PrHKT1;2 (Figure 8a,b), and the yeast growth was inhibited even under control conditions containing low Na^+^ levels.

## 3. Discussion

### 3.1. Transcriptomic Profiles Reveal the Interspecific Difference in Salt Responsiveness between the Two Populus 

Our transcriptome data comprehensively depict the salt response profiles of the two *Populus* under either short- (24 h) or long-term (12 d) salinity stress; the reliability of the data was verified by qRT-PCR (Figure S4). Our data reveal the distinct strategies of the two *Populus* responses to the short-term salt stress. The up-regulated DEG numbers substantially surpassed the downregulated in *P. alba*, whereas in *P. russkii* they were much more comparable (Figure 3b). Numerous DEGs detected between *P. alba* and *P. russkii* in the control and up-regulated genes (*P. russkii* versus *P. alba*) are mainly involved in the generation of pre-metabolites and energy, transport, and carbohydrate metabolic processes (Figure 5b). This energy reserve endows *P. alba* with a higher defense response to salt in the short term. Under long-term salt stress, phenotypic observation indicates that *P. alba* was able to resist salinity to keep growing, whereas the *P. russkii* leaves were severely dehydrated and numerous genes involved both in multiple metabolic (GO:0009056; 0019748; 0006629) and cellular processes were downregulated (Figure 3c). In contrast, in *P. alba* the up-regulated DEGs were three times as much as the downregulated, these genes being significantly enriched in response to biotic stimulus (GO:0009607) and carbohydrate metabolic (GO:0005975) and catabolic processes (GO:0009056), which suggests that *P. alba* effectively mobilizes a series of defenses and energy to combat salt stress. This response to salinity is highly similar with that of *P. euphratica*, a relative species of *P. alba* [18,19].

The transcriptomic data identified many salt-tolerant candidate genes in poplars, including among them some important transcription factors previously reported under either osmotic stress or salt stress, i.e., RD26a/ANAC072, bZIP1, MYB78, MYB43, WRKY48, and GAPC2 (Figure 4c) [29,31,32], as well as consisting of several protein-encoding genes such as DI21, ABA-induced PP2C (HAI1)/SAG113, and the LEA4-5 which related to osmotic stress [30]. These genes maybe contribute to improve salt tolerance in forest and crop plants.

### 3.2. Interspecific Difference in Poplar Salt Tolerance Is Related to Na^+^ Transportation Capacity and to Its Regulation

Previous studies show that high tolerance to salinity is related to a lower Na^+^ concentration accumulated in shoots or leaves as well as to K^+^/Na^+^ homeostasis in plants [10,18,45]. In our study, pharmacological analysis reveals that the capacity of Na^+^ uptake in *P. alba* via K^+^ inward rectifier channels and HKT transporters is higher than that in *P. russkii* (Table 1). The transcription of the K^+^ inward rectifier channel genes, such as AKT2, AKT5, KT1, KT2a/b, and KT12, were steady expressed in *P. alba*, but their expression was reduced in *P. russkii* under salt stress (Figure 7). The downregulation of these genes contributes to a decreased Na^+^ uptake and a decreased K^+^ uptake, producing the negative effect of maintaining a high K^+^/Na^+^ cytoplasmic ratio, thereby declining salt tolerance. The HKT transporter function in Na^+^ uptake in roots plays a key role in Na^+^ translocation and K^+^/Na^+^ homeostasis maintenance in xylem parenchyma cells [14,15,16,21]. In *P. trichocarpa*, there is one *HKT1* ortholog gene together with a *HKT1*-like pseudogene. However, in the desert poplar *P. euhratica*, the gene has expanded to four members [20], indicating it plays a functional role in salt tolerance for poplar plants. In this study, we found there were two ortholog genes of *HKT1*, i.e., *HKT1;1* and *HKT1;2*, in both *P. alba* and *P. russkii*. We detected only *HKT1;1* expressed in the two *Populus* roots and its transcription decreased under salt stress, which serves as a defense strategy in response to salinity. *HKT1;2*, an orthology gene of *HKT1;1*, was mainly expressed in *Populus* leaves and plays a critical role in Na^+^ transport/translocation in photosynthetic apparatus. As the yeast analysis shows, PaHKT1;2 presents a higher Na^+^ transportation activity than PrHKT1;2, suggesting that PaHKT1;2 might play an important function in Na^+^ transportation/translocation or salt tolerance in *P. alba*. Extensive SNP variations detected with the *HKT1;2* gene coding sequence between the two *Populus* also suggest that the HKT allele of *P. alba* has a superior performance than *P. russkii* and might have a significant role in salt tolerance; therefore, its function needs further research.

Na^+^ uptake via the NSCCs pathway was dominant in *P. russkii* under high salinity rather than in *P. alba* (Table 1). Gd^3+^ and La^3+^ serve as the broad-spectrum inhibitors; although they can block NSCCs, they also play important roles in inhibiting Ca^2+^ channels [46]. The changes in Ca^2+^ transportation can directly impact Na^+^ and K^+^ channels via signal transmission [10,45,47]. The uptake of Na^+^ mediated by NSCCs depends on the external Na^+^ concentration and can be—to a lesser extent—in competition with K^+^, suggesting that NSCCs may serve as a passive way to mediate Na^+^ absorption. In this study, the NSCCs play a major role in LATS Na^+^ uptake in *P. russkii*, which explains the weak resistance to high salinity.

The distribution of Na^+^ in the cells and its translocation in different organs are critical to plant salt tolerance when it enters into root cells. Compartmentalizing Na^+^ into vacuoles via the Na^+^/H^+^ antiporter NHX1 and squeezing out the protoplast by SOS1 is a very important strategy for Na^+^ detoxification in plants [1,23,48,49]. Our results indicate that the transcription of *NHX1* and *SOS1a* can maintain a high level in *P. alba* while maintaining low levels in *P. russkii* under salt stress; this is another explanation for *P. russkii* being highly sensitive to salt stress. 

### 3.3. Phytohormone-Regulated Responses Are Related to Plant Salt Tolerance

In this study, the transcriptions of NCED3 and ACO1 as rate-limiting enzymes in abscisic acid (ABA) and ethylene biosynthetic pathways were significantly downregulated in *P. russkii* but up-regulated in *P. alba* under long-term salt stress, respectively. Correspondingly, the ethylene response factors *ERF6, ERF71,* and *HRA1* were also significantly decreased in *P. russkii* but up-regulated in *P. alba* (Figure 6b). The expression patterns of these genes shows that *P. alba* presents a higher level of ethylene and ABA syntheses than *P. russkii*. Previous studies demonstrate that ethylene improves plants’ salt tolerance, mainly via inducing antioxidant defense and ROS detoxification and by maintaining K^+^/Na^+^ homeostasis [50,51]. The *ERF6, ERF71,* and *HRA1* factors were reported to play an important role in resistance to abiotic stresses in *Arabidopsis* and crops plants [37,52,53]. ABA modulates plants responses to salt stress largely through osmotic regulation and by inducing stomatal closure. Therefore, the differences in both ethylene and ABA syntheses between *P. alba* and *P. russkii* are identified here as critical factors that cause the differential salt resistance under long-term salt stress. 

GA2-oxidases (GA2oxs) regulate the deactivation of bioactive GAs and are very important to plant development and stress responses [54]. *GASA* (*Gibberellic Acid Stimulated in Arabidopsis*) genes encoding cysteine-rich peptides are involved in plant development and environmental adaption [55]. It has been reported that overexpression of *GA2ox* and *GASA14* genes in *Arabidopsis* might enhance plants’ resistance to high salinity [56,57]. In our study, the *GA2ox3*, *GA2ox6,* and *GASA14s* maintained a steady high expression in *P. alba* but decreased in *P. russkii* under long-term salt stress (Figure 6c), which indicates the GAs’ metabolism in *P. alba* differs from that in *P. russkii*. Furthermore, we found many genes in auxin biosynthetic or signaling pathways, such as *IAAs, ARFs, SAURs,* and *PINs*, showing a reverse expression pattern between *P. alba* and *P. russkii* (Figure 6c). Aux/IAA, ARF, and SAURs are key regulators of auxin responses that also play important roles in plant development and their responses to environmental cues [58,59,60]. A recent study shows that the heterologous expression of grapevine *VvIAA18* in tobacco can significantly enhance salt tolerance [61]. In this study, these auxin response genes presented a steady differential expression between the two *Populus* and were hardly affected by salinity, indicating they are mostly regulated by auxin concentration rather than directly responding to salt stress. The expression patterns of these genes reveals that the differential local auxin levels between the two *Populus* is one of the key factors shaping its salt tolerance. 

Under salt stress, the activities of POD, APX, and GR increased in *P. alba* but did not significantly change in *P. russkii*, and their encoding genes had a higher expression level in *P. alba* than in *P. russkii* (Figure S3), which underlines the transcriptional regulation effects on enzyme activities resulting from protein abundance. The transcription of *galactinol synthase1* (*GolS1*) and *GolS4* genes increased in *P. alba* but were diminished in *P. russkii* under salt stress. GolS is a key enzyme catalyzing the biosynthesis of raffinose family oligosaccharides (RFOs) in plants [38]. Overexpressed *GolS* can significantly enhance the tolerances to salt, cold, and drought stresses in poplar and cucumber [62,63,64]. In addition, most of flavonoid- or anthocyanin-related biosynthetic genes maintained a high expression under long-term salt stress in *P. alba* but were downregulated in *P. russkii*. Many studies show that overexpression of these genes can improve salt resistance [65,66]. In contrast, laccases (LACs) genes had higher expression levels in *P. russkii* than those in *P. alba* in control, whereas under long-term salt stress they were downregulated in *P. russkii* although they did not change in *P. alba*. These genes are involved in lignin synthesis and their transcription is positively related to their growth rate [67,68]. Although previous studies have found that the growth rate of *P. russkii* was higher than *P. alba* under normal conditions, its salt or drought tolerance was weaker than *P. alba* [25,27]. The expression pattern of *LACs* explains this and also reveals the different opportunistic approaches to trade-offs between growth and defense in these two poplar species: *P. russkii* adopts an opportunistic growth strategy while *P. alba* adopts an opportunistic defense strategy.

This work reveals the molecular mechanism underlying the differential salt-tolerance between two poplar species under salinity stress. Compared to the salt-sensitive poplar (*P. russkii*), the salt-tolerant poplar (*P. alba*) shows a high transcriptional level of cellular energy metabolism processes, thus effectively initiating the stress defense responses under salt stress. Moreover, PaHKT1;2 has a strong Na^+^ transportation ability that effectively recycles xylem Na^+^ transport in *P. abla* under salt stress. On a physiological level, it is the higher antioxidant enzyme activities, osmoregulation, and stress-related hormone levels (ABA and ethylene) that altogether confer *P. alba* high salt tolerance. This study has significant implications for improving salt resistance in crop plants.

## 4. Materials and Methods

### 4.1. Plant Material and Experimental Treatment

One-year-old saplings of *P. alba* and *P. russkii* were collected at their natural habitat (44.29° N, 87.93° E, 200 m altitude) in Xinjiang, Northwest China. The stems of *P. alba* and *P. russkii* were cut into 20 cm-long pieces (diameter with 1 cm) and grown within a hydroponic culture with a half-strength Hoagland solution containing 2.5 mM KNO_3_, 2 mM Ca(NO_3_)_2_·4H_2_O, 1 mM MgSO_4_·7H_2_O, 0.5 mM KH_2_PO_3_, 25 μM H_3_BO_3_, 1.25 μM KI, 25 μM MnSO_4_·4H_2_O, 7.5 μM ZnSO_4_·7H_2_O, 0.25 μM Na_2_MoO_4_·2H_2_O, 0.025 μM CoCl_2_·6H_2_O, 0.025 μM CuSO_4_·5H_2_O, and 25 μM Fe(III)-Ethane-1,2-diyldinitrilo tetra-acetic acid (EDTA). The hydroponic culture solution was refreshed every 3 days. An air pump supplying air to plant roots was installed for the hydroponic water culture. The cuttings were cultivated inside a controlled climate chamber at 25/18 °C with a 14/10 h light/dark photoperiod with a maximum photon flux density of 400 μmol m^−2^ s^−1^ (photosynthetically active radiation) supplied by fluorescent lights; the relative humidity was within a 65~70% range. The cuttings were rooting and sprouting after a week.

Four weeks after the start of the hydroponic culture, we selected sprouting cuttings with uniform shoots, with an average height of 32 cm for *P. alba* and 35 cm for *P. russikii*, and then initiated the experiment. The treatments included both a control (CK) and a salt stress group (with 100 mmol L^−1^ NaCl) for each of the two *Populus*. The control was cultured in the Hoagland solution without NaCl, whereas the salt stress treatment group was cultured inside a Hoagland solution containing 100 mmol L^−1^ NaCl. Each treatment group in both *Populus* species consisted of three biological replications (six plants per replicate). The fourth to sixth fully expanded leaves of the treatment group and control were collected for ion content determination and experimental analyses. Intact roots of the salt stress treatment group and control were collected and frozen immediately using liquid nitrogen and subsequently stored at −80 °C for RNA-sequencing and physiological experimental analyses. 

### 4.2. Measurement of Na^+^ and K^+^ Content in Poplar Leaves

Na^+^ and K^+^ concentration in the two *Populus* species were measured with an atomic absorption spectrophotometer (Perkin-Elmer, AA700, Waltham, MA, USA) using the method described previously [19]. Leaves of the salt-treated and control plants were oven-dried at 65 °C during more than 3 days to achieve complete dehydration. The dry samples were ground into fine powder (100 mg) and incubated into a 10 mL 0.5 M HNO_3_ solution subjected to shaking extraction for 48h at room temperature. Afterwards, we used centrifugation at 12,000 rpm for 10 min to settle the solid material down into the tube bottom, and then we transferred the extracts and diluted them with deionized H_2_O (Milli-Q, Merck KGaA, Darmstadt, Germany) to measure the Na^+^ or K^+^ content.

### 4.3. Determination of Antioxidant Enzymes Activities, Glycine-Betaine and Malondialdehyde (MDA) Content

Antioxidant enzymes (POD, CAT, APX, and GR) activities were assayed as described in our previous study [27]. Fresh leaves (0.5 g) were ground in liquid nitrogen and extracted with a 50 mM potassium phosphate buffer (pH 7.8) containing 0.1 mM EDTA, 1% (*w*/*v*) polyvinyl pyrrolidone (PVP), 0.1 mM phenylmethane sulfonyl fluoride (PMSF) solution, and 0.2% (*v*/*v*) Triton X-100. POD (EC1.11.1.7) activity was measured at 470 nm as described in [69]. CAT (EC1.11.1.6) activity was assayed as described in [70]. APX (EC1.11.1.11) activity was determined as described by [71]. GR (EC1.6.4.2) activity was measured using a glutathione reductase activity kit (Cominbio, Suzhou, China, Cat.# GR-2-W) following the manufacturer’s instructions. Absorbance at 340 nm was monitored by spectrophotometer. 

Glycine-betaine content was measured using a plant Betaine content kit (Cominbio, Suzhou, China, Cat.# TCJ-2-G) following the manufacturer’s protocol. Absorbance was measured at 525 nm and expressed as μg g^−1^ dry weight (DW). Cellular membrane lipid peroxidation analysis was based on MDA concentration according to our previous study [27]. Absorbance was measured at 450 nm, 532 nm, and 600 nm using a spectrophotometer. MDA concentration was calculated using the formula: MDA (μmol L^−1^) = 6.45 (A_532_ − A_600_) − 0.56A_450_.

### 4.4. RNA Extraction, cDNA Library Preparation, and RNA Sequencing 

Total RNA was extracted from the poplar roots using an RNeasy plant mini kit (Qiagen, Hilden, Germany) following the manufacturer’s protocol. Each treatment group or control sample had three biological replicates. RNA concentration and purity was measured using a Qubit 2.0 fluorometer (Invitrogen, Waltham, MA, USA). High-quality RNA was processed for RNA-seq library construction. Two micrograms of total RNA were used for mRNA isolation, while mRNA fragmentation and cDNA library construction were conducted using a TruSeq Stranded mRNA Library Prep Kit (Illumina, San Diego, CA, USA) as stipulated by the manufacturer’s instructions. The index codes were added to attribute sequences to each sample. The cDNA libraries were sequenced at the Beijing Genomics institution (BGI, Shenzhen, China) on the Illumina HiSeq 2500 System by 50 bp single-read sequencing.

### 4.5. Analysis of RNA-Seq and Identification of DEGs 

RNA-seq raw reads were filtered to remove adapter sequence and low-quality reads by Trimmomatic (version 0.36) software. The clean reads were aligned to the *P. trichocarpa* reference genome (v4.1, PhytozomeV13) using HISAT2 (version 2.1.0). Gene expression quantification and differentially expressed genes (DEGs) calculation were conducted by Cufflinks (version 2.2.1) with default parameters [72]. DEGs were identified between two comparisons using the following criteria: |log2 (fold change)| > 1 and false discovery rate (FDR) < 0.01. The FDR was generated from an adjusted *P*-value using the Benjamini-Hochberg method. GO enrichment and heatmap visualization were generated using TBtools (version 1.086) [73].

### 4.6. Reversal Transcription and qRT-PCR 

Four micrograms of total RNA was used to synthesize first-strand cDNA with a reversal transcription reagent kit (TaKaRa). Synthesized cDNA was diluted to a final volume of 100 μL, and 1 μL was added as the template for detection in each reaction. qRT-PCR analyses were conducted using SYBR Premix ExTaq (TaKaRa) with a CFX96 real-time system (Bio-Rad Laboratories, Inc., Hercules, CA, USA). *Populus* elongation factor-1 alpha (EF-1-α) was selected as the internal control to normalize gene transcription. Three biological replicates were performed for each qRT-PCR analysis. The levels of gene expression were evaluated by calculating the threshold cycle (Ct) based on the 2^−ΔΔCt^ method and were measured in terms of relative quantitative variation.

### 4.7. Pharmacological Analysis of Low-Affinity Na^+^ Transport 

After four weeks of hydroponic cultivation, sprouting cuttings of *P. alba and P. russkii*—whose shoots had heights of 32 cm and 35 cm—were used to evaluate the effect of ion channel/transporter inhibitors on Na^+^ uptake/transport via the low-affinity transport system. The applied inhibitors in this study included K^+^ channel blockers (TEA-Cl, CsCl, and BaCl_2_), and non-selective cation channel (NSCCs) blockers (LaCl_3_ and GdCl_3_). The concentration of inhibitors was determined based on the following previous studies: [74] with 10 mM TEA-Cl; [44] with 10 mM CsCl; [75] with 5 mM BaCl_2_; and [44] with 50 μM LaCl_3_ and 50 μM GdCl_3_. The inhibitor was added to the hydroponic culture solution containing 100 mM NaCl, taking the salt stress treatment group (i.e., 100 mM NaCl without any inhibitors) as the control. Each treatment had three replications and each replicate had three plants. The inhibitors treatment lasted 72 h and Na^+^ and K^+^ concentrations in the treated plants leaves were measured at 0 h (before treatment), 8 h, 24 h, 48 h, and 72 h. 

### 4.8. Yeast Transformation and Growth Analysis

The yeast (*Saccharomyces cerevisiae*) strain G19 (Matα ura3 his3 trp7 ade2 ena1Δ::HIS3::ena4Δ) [76] was used to analyze the Na^+^ transporting function of HKT1;1 and HKT1;2 in the two *Populus*. The G19 strain was transformed with the pYES2 plasmid alone (empty vector) or by pYES2 containing the coding sequences of *PaHKT1;1*, *PrHKT1;1*, *PaHKT1;2*, and *PrHKT1;2*. Transformants were cultured on a synthetic dropout medium without uracil (SD/-Ura), and with 2% glucose as the carbon source for selection and normal growth, or with 2% galactose for inducible expression of HKT genes. Complementary analysis was performed by inoculation of 10 μL drops of cell suspension (OD600 = 1 or its dilution series) onto the surface of SD/-Ura/galactose agar medium supplied with 0, 50, 100, 150, or 200 mM NaCl. The results were recorded after 2 days of incubation at 30 °C. As for the growth curve test, yeast cells were incubated in SD/-Ura/galactose liquid medium with or without 100 mM NaCl at 30 °C with shaking. The absorbance was measured at 600 nm every 4 h. 

### 4.9. Statistical Analysis

Both datasets were separately analyzed using SPSS software (v19.0), each bar representing the mean ± SE of at least three replicates. Different letters above the bars indicate significant differences, and values of *p* < 0.05 represented statistical significance using LSD’s test.

## Figures and Tables

**Table 1:** Effects of pharmacological treatments on low-affinity Na^+^ influx in *Populus* plants under 100 mM NaCl. Na^+^ concentrations were measured before treatments and after NaCl and inhibitor treatments for 24 h (each treatment had three biological replicates). Measurements significantly different from the control are indicated with a *, *p* < 0.05 (*t*-test). Values represent mean ± SD (n = 3).

Treatment (Meaured at 100 mM NaCl)	*P. russkii*		*P. alba*	
	Na^+^ Influx (nmol g^−1^ DW h^−1^)	Suppression	Na^+^ Influx (nmol g^−1^ DW h^−1^)	Suppression
NaCl (100 mM)	148.44 ± 7.85	0	84.02 ± 3.94	0
TEA^+^ (10 mM)	38.56 * ± 4.1	74.02%	5.72 ± 0.16	93.19%
Cs^+^ (10 mM)	33.15 * ± 2.31	77.67%	8.57 ± 0.62	89.79%
Ba^2+^ (5 mM)	32.87 * ± 3.45	77.85%	0.76 ± 0.26	99.10%
Gd^3+^ (50 μM)	29.24 * ± 2.03	80.03%	9.67 ± 0.29	88.49%
La^3+^ (50 μM)	32.63 * ± 3.47	78.01%	8.33 ± 1.49	90.09%

## Data Availability

The RNA-seq data generated in this study were deposited in the NCBI Sequence Read Archive (BioProject ID: PRJNA896337; https://www.ncbi.nlm.nih.gov/bioproject/PRJNA896337, accessed on 10 January 2023). The datasets supporting the conclusions of this article are included within the article and its additional files.

## Supplementary Materials

The supporting information can be downloaded at: https://www.mdpi.com/article/10.3390/ijms24065732/s1.
