# Supplementary material for: Transcriptome and Low-Affinity Sodium Transport Analysis Reveals Salt Tolerance Variations between Two Poplar Trees
Source: Int J Mol Sci. 2023 Mar 17;24(6):5732. doi: 10.3390/ijms24065732 (PMC10058024; doi:10.3390/ijms24065732)
Supplement: Supplementary file 1 [file ijms-24-05732-s001.zip › Supplementary figures& tables.pdf]

Supplementary Figures

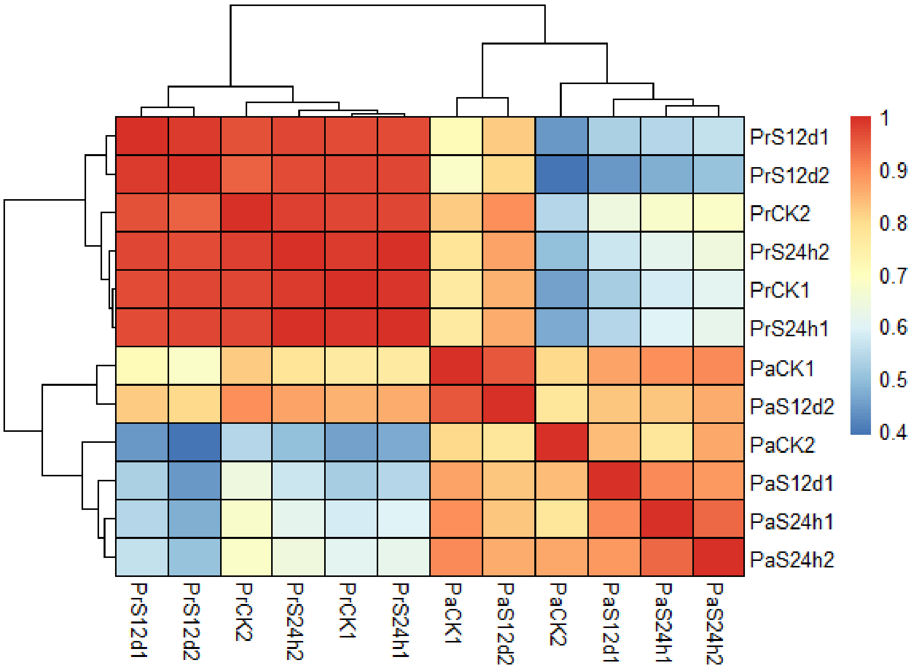

Figure S1. Correlation matrix heatmap of RNA-seq replicates of *P. russkii* (Pr) and *P. alba* (Pa) control (CK), short-term (S24h) and long-term (S12d) salt stress samples. Pearson correlation coefficient was calculated by RPKM. Two biological replicates are shown.

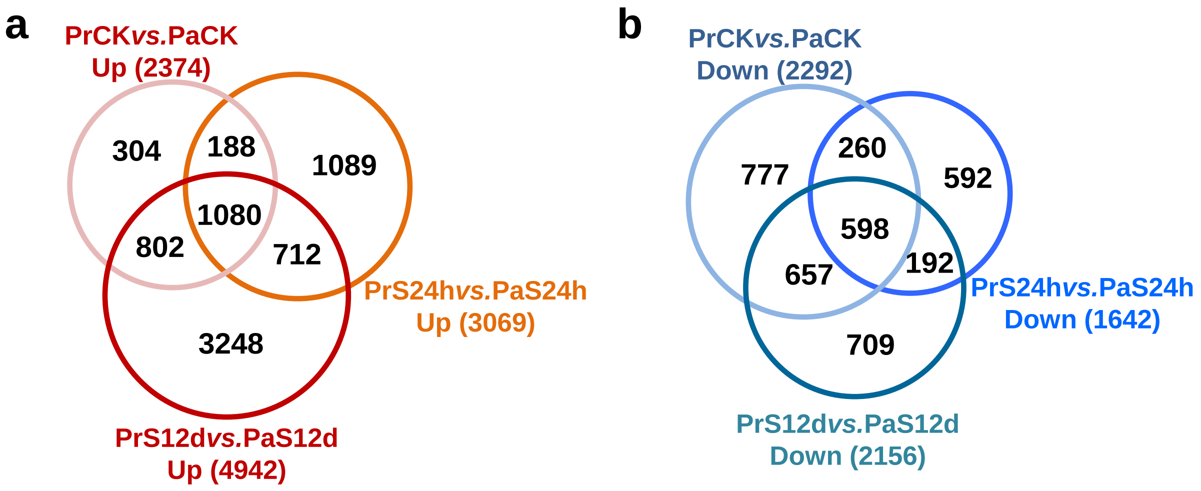

Figure S2. Venn diagrams showing the conservatively **(a)** up-regulated or (**b**) downregulated genes in *P. alba* versus *P. russkii*.

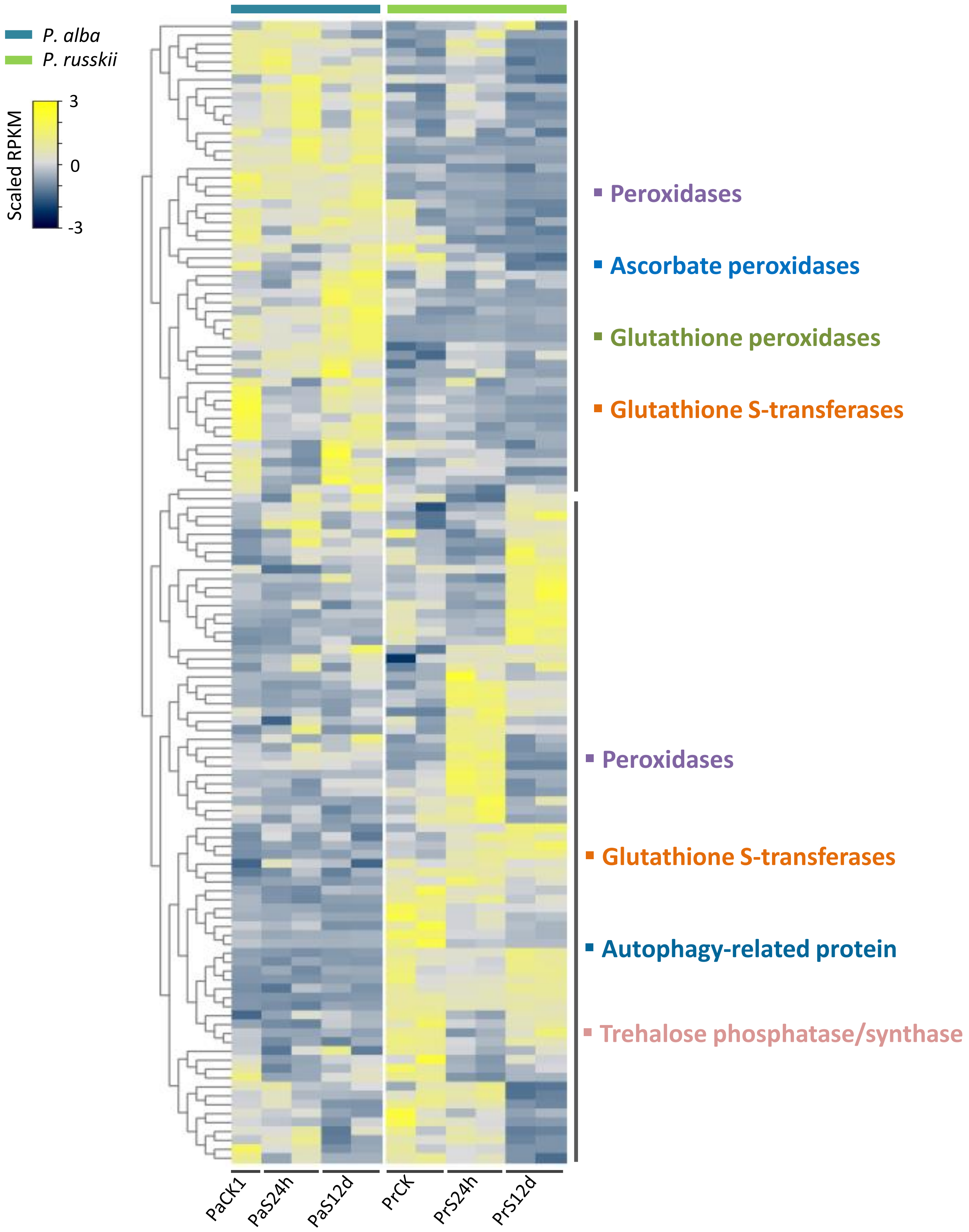

Figure S3. Heatmap showing differential expression patterns of the genes involved in redox processes of the two *Populus* species under salt stress.

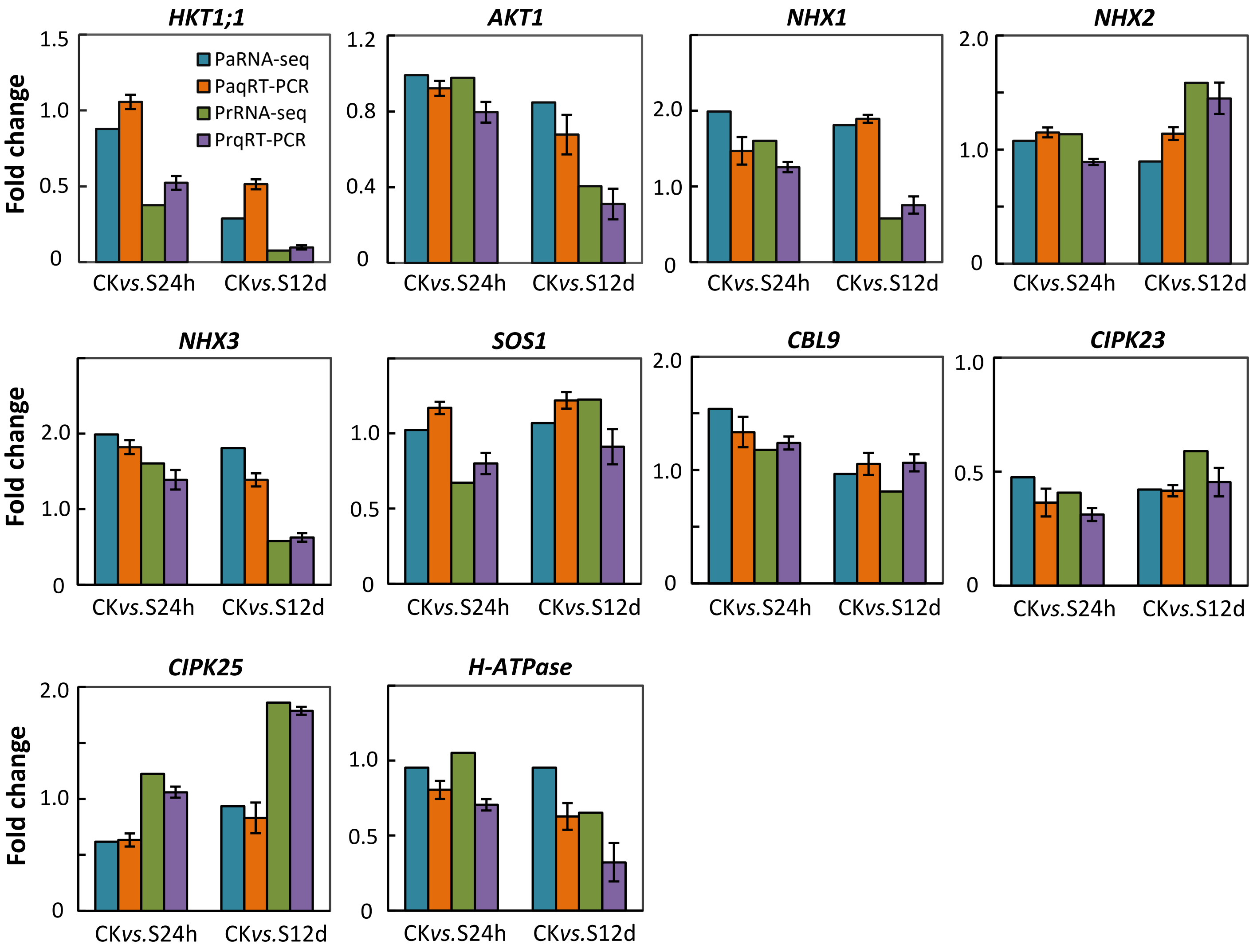

Figure S4. Quantitative real-time PCR (qRT-PCR) and RNA-seq analysis of the expressions of Na^+/K^+ transporter and calcium-binding protein encoding genes in roots of *P. alba* and *P. russkii* under salt stress. qRT-PCR data shown are the mean ± SD of three replicates (n = 3).

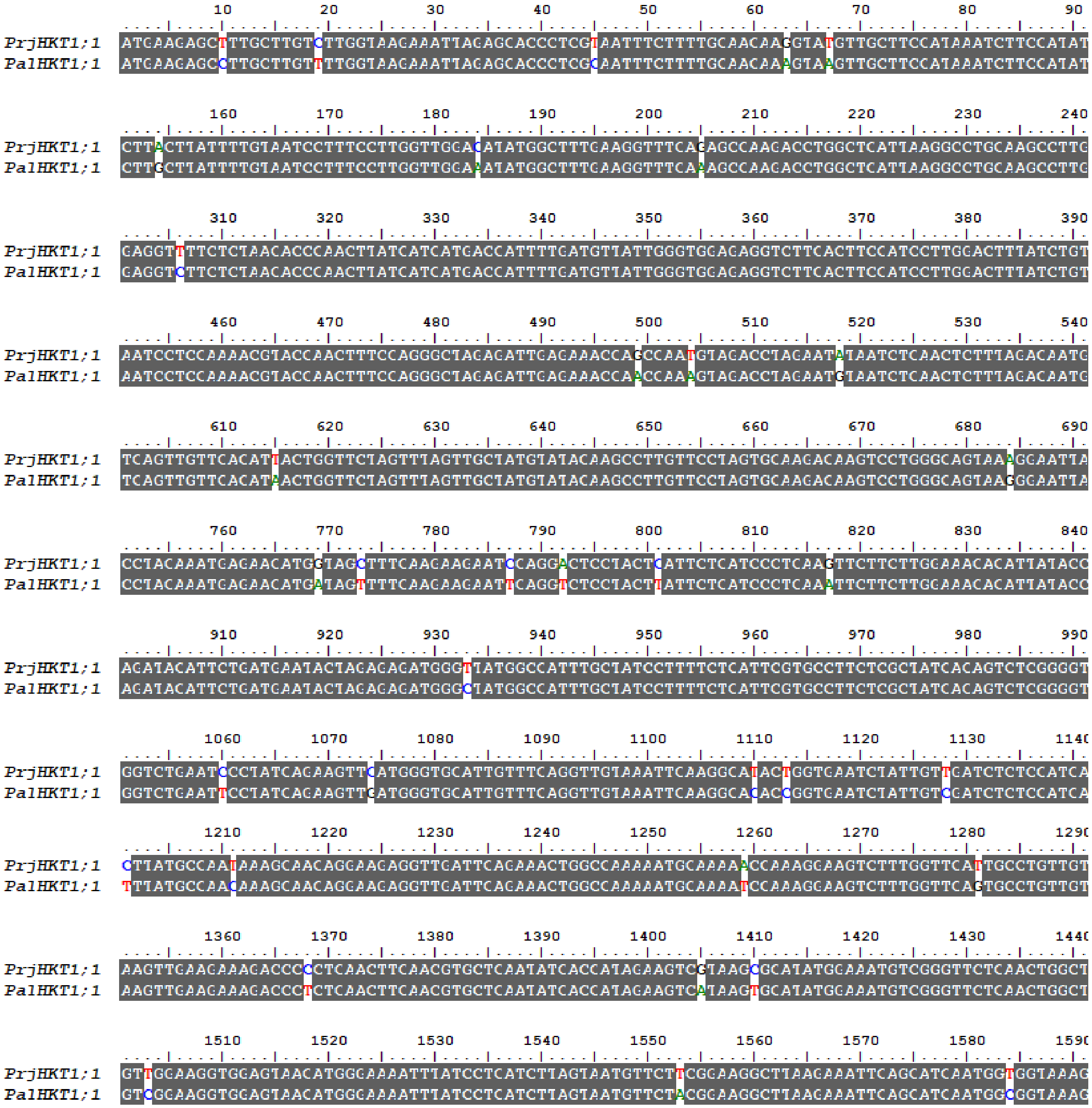

Figure S5. The alignment of *HKT1;1* gene coding sequence showing the extensive nucleotide variations between *P. russkii* (Prj) and *P. alba* (Pal).

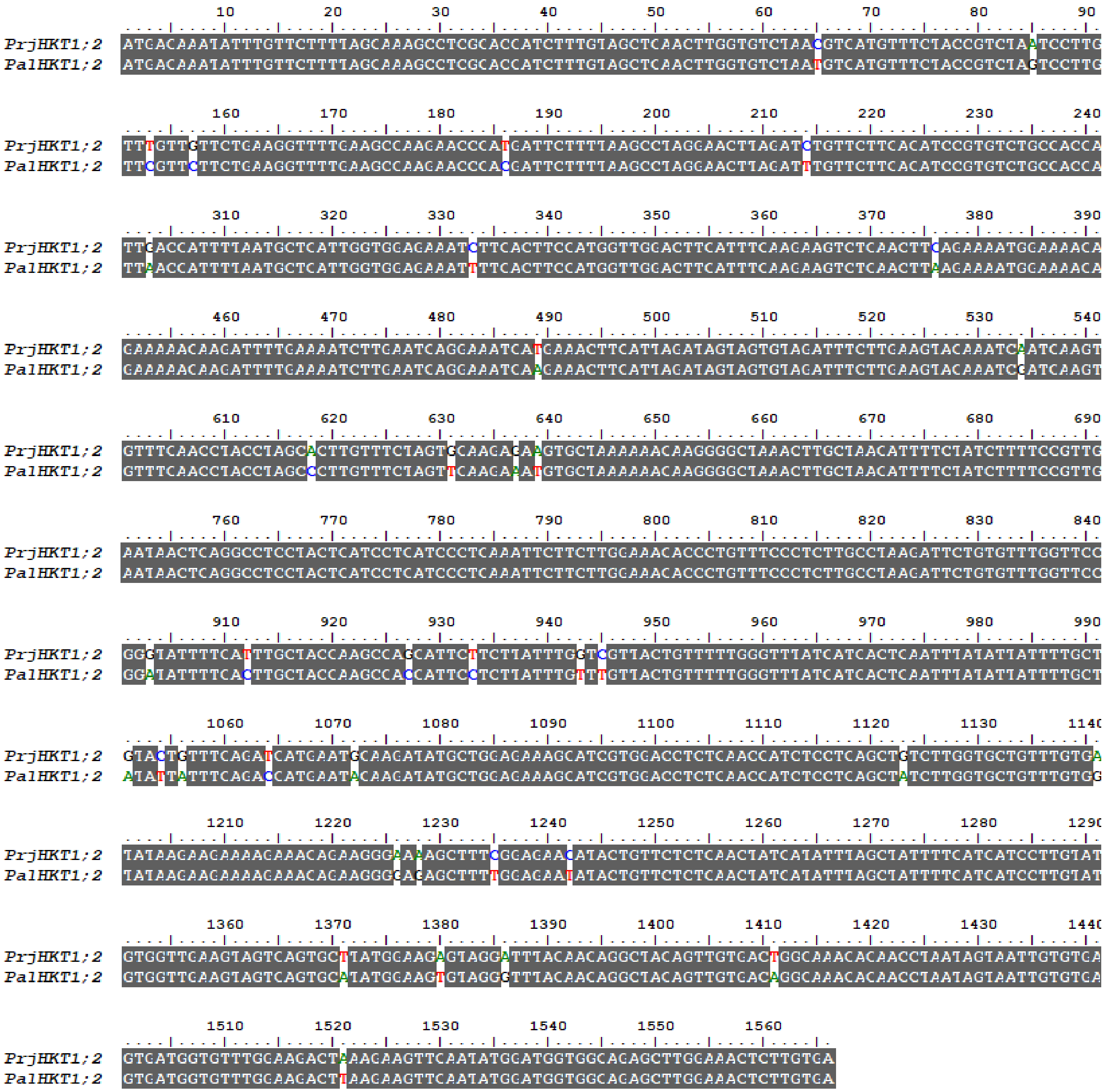

Figure S6. The alignment of *HKT1;2* gene coding sequence showing the extensive nucleotide variations between *P. russkii* (Prj) and *P. alba* (Pal).

Supplementary Tables

Table S1. Summary of the alignment information of the transcriptome data.

Treatments	Replicates	Clean reads	Total aligned reads	Uniquely aligned reads	Multiple aligned reads	Transcripts with RPKM > 1
PaCK	1	8,256,840	5,019,333 (60.79%)	4,640,928 (56.21%)	378,148 (4.58%)	22,537
PaCK	2	8,547,701	5,226,064 (61.14%)	4,818,441 (56.37%)	407,547 (4.77%)	20,834
PaS24h	1	8,973,433	5,450,463 (60.74%)	5,060,257 (56.39%)	389,958 (4.35%)	21,907
PaS24h	2	8,965,375	5,424,948 (60.51%)	5,036,256 (56.17%)	388,665 (4.34%)	22,034
PaS12d	1	9,005,119	5,298,612 (58.84%)	4,892,459 (54.33%)	406,149 (4.51%)	22,336
PaS12d	2	7,847,916	4,833,531 (61.59%)	4,462,683 (56.86%)	370,827 (4.73%)	22,739
PrCK	1	8,479,539	6,516,526 (76.85%)	6,049,622 (71.34%)	467291 (5.51%)	23,018
PrCK	2	9,100,053	6,774,989 (74.45%)	6,295,245 (69.18%)	479567 (5.27%)	23,262
PrS24h	1	7,916,670	6,011,919 (75.94%)	5,603,003 (70.77%)	408782 (5.16%)	23,143
PrS24h	2	8,705,226	6,522,826 (74.93%)	6,077,078 (69.81%)	445652 (5.12%)	23,254
PrS12d	1	9,798,656	7,189,274 (73.37%)	6,688,671 (68.26%)	500760 (5.11%)	21,741
PrS12d	2	8,234,221	6,058,740 (73.58%)	5,640,570 (68.50%)	418329 (5.08%)	21,881

Table S2. Primers using for the qRT-PCR analysis.

Primer name	Sequence
PtrHKT1;1-F	CAGTCCTTCCTTCGTGTGCT
PtrHKT1;1-R	TTGCAGGCCTTAATGAGCCA
PtrAKT1-F	TGTTGCTGGAGACGGAGAAC
PtrAKT1-R	TGCATTGCTGACCTCCCATT
PtrNHX1-F	TGCAACTGCAAGTTAGAGGGT
PtrNHX1-R	AATGCAAGCACAAAGGAGCG
PtrNHX2-F	TCAGCACAGTGGTGTTTGGT
PtrNHX2-R	CTAAGGAGTCCTGCCCCTCT
PtrNHX3-F	GCTGTTTTATCATATCACTAGGTGC
PtrNHX3-R	CATTTACGACACCCTCCCCG
PtrSOS1-F	CTCATGGGAGCACACTAGGC
PtrSOS1-R	TGCACTTTCCTGCCAAAGGA
PtrCBL9-F	ACTCTCCCCTACTTGAGGGAC
PtrCBL9-R	CACTTCCCCCTCTATGCAGC
PtrCIPK23-F	TCAAGGCTGACTTCGCATGT
PtrCIPK23-R	CCACATCGTCGAGACCAACA
PtrCIPK25-F	GCGCCTTTGGGTTTTGATGT
PtrCIPK25-R	TCTCCACCAGACTTGCGAAC
PtrATPase-F	TGCATTGGCAAATGGAAGTGG
PtrATPase-R	AGCATTGCCAGCATTGTTTTCT
PtrEF1-α-F	TCCGTCTTCCACTTCAGGATGTCT
PtrEF1-α-R	GTCACGACCATACCAGGCTTCAG
